# Smallpox in the Post-Eradication Era

**DOI:** 10.3390/v12020138

**Published:** 2020-01-24

**Authors:** Hermann Meyer, Rosina Ehmann, Geoffrey L. Smith

**Affiliations:** 1 Bundeswehr Institute of Microbiology, 80937 Munich, Germany; 2 Department of Pathology, University of Cambridge, Cambridge CB2 1QP, UK; gls37@cam.ac.uk

**Keywords:** smallpox, variola virus, vaccination, antiviral, eradication

## Abstract

Widespread vaccination programmes led to the global eradication of smallpox, which was certified by the World Health Organisation (WHO), and, since 1978, there has been no case of smallpox anywhere in the world. However, the viable variola virus (VARV), the causative agent of smallpox, is still kept in two maximum security laboratories in Russia and the USA. Despite the eradication of the disease smallpox, clandestine stocks of VARV may exist. In a rapidly changing world, the impact of an intentional VARV release in the human population would nowadays result in a public health emergency of global concern: vaccination programmes were abolished, the percentage of immunosuppressed individuals in the human population is higher, and an increased intercontinental air travel allows for the rapid viral spread of diseases around the world. The WHO has authorised the temporary retention of VARV to enable essential research for public health benefit to take place. This work aims to develop diagnostic tests, antiviral drugs, and safer vaccines. Advances in synthetic biology have made it possible to produce infectious poxvirus particles from chemicals in vitro so that it is now possible to reconstruct VARV. The status of smallpox in the post-eradication era is reviewed.

## 1. History of Smallpox

Smallpox is caused by the variola virus (VARV), a member of the *Orthopoxvirus* (OPV) genus of the *Poxviridae*. In the past, the disease was named smallpox because the lesion size was smaller than that of great-pox (*syphilis*). Edward Jenner described smallpox as “the most dreadful scourge of the human species.” Exact numbers of deaths are not recorded but estimates amount to 400 million people in the 20th century alone [1]. The origin of VARV remains unknown; the first clinical descriptions of smallpox were noted in China (4th century), in India (7th century), and in the Mediterranean area (10th century). Several Egyptian mummies have skin lesions that resemble smallpox. Before the 15th century, smallpox was observed in Europe and Asia. However, during colonialism smallpox was imported by the Europeans into the Americas, Southern Africa, and Australia between the 15th and 18th century. Confronted with a novel pathogen, the immunologically naïve indigenous populations of these continents suffered from high case-fatality rates in smallpox outbreaks with major historical consequences. Towards the end of the 19th century, a less lethal form of smallpox was documented in South Africa and the United States and later on in South America. These viruses called amass, alastrim, and kaffir were designated variola minor to differentiate them from variola major, the causative agent of classical smallpox. The case-fatality rate for variola minor was only 1%, which is in sharp contrast to 30% for variola major. 

## 2. Clinical Features of Smallpox

The variola virus infection is transmitted through aerosol. This was proven in a hospital building in Meschede, Germany, where a total of 17 smallpox cases were infected by virus particles disseminated by air over a considerable distance [2]. Usually, the virus enters the body by the inhalation of microdroplets shed from the respiratory tract of infected persons [1]. This occurs when persons have face-to-face contact with patients during the first 7 days of overt disease. The virus also infects through contact with skin, body fluids, or pock material. After infection and an incubation period of approximately 7 to 19 days, patients experience a prodromal phase with high fever, malaise, and headaches. The virus replicates in mucosal membranes and is released from ulcerative lesions even before other, more characteristic symptoms occur. The most striking feature of smallpox is a rash leading to the well known cutaneous lesions. The rash starts in different parts of the body and—after passing through the stages of macules, papules, and vesicles—scabs are formed that eventually desquamate during the following 2–3-week period resulting in the formation of the classic pock scars. Smallpox lesions range from 0.5–1 cm in diameter, they are deep-seated in the epidermis and progress through the same stages synchronously.

Smallpox, as a disease, was relatively easy to recognize with its characteristic skin lesions, however, the following exanthematous illnesses have to be considered as differential diagnosis: severe chickenpox rash, monkeypox, generalized vaccinia virus or cowpox virus infections, eczema vaccinatum, disseminated varicella-zoster, herpes simplex virus infections, drug reactions, erythema multiforme, enteroviral infections, rickettsialpox rash, secondary syphilis, scabies, insect bites, impetigo, and molluscum contagiosum [3]. Diseases confused with haemorrhagic smallpox included acute leukaemia, meningococcaemia and idiopathic thrombocytopenic purpura. The US Centers for Disease Control and Prevention (CDC) have developed an algorithm for patient evaluation [4,5] to assist physicians and health care workers. A suspected case of smallpox must be reported immediately. Work with infectious VARV can only be performed in two biosafety level 4 laboratories sanctioned by the WHO. 

## 3. The History of Smallpox Vaccination

It was understood early in the history of smallpox that persons who survived an infection never developed the disease again. Additionally, it became clear that many individuals infected by skin lesions suffered a less severe form of smallpox than those infected by the respiratory route. This led to an early immunization procedure that became known as variolation: immunologically naïve persons were infected via cutaneous scratches with pustular fluid/material collected from smallpox patients. Initially developed in India and China, it became common in Europe and its colonies in the 18th century. Despite its success, the number of patients that still developed the classical form of smallpox after variolation with the full clinical picture and high mortality rate was not negligible. In the late 18th century, it was known that milkmaids, who had contracted a zoonotic infection, cowpox, usually survived smallpox [6]. Based on these observations, Edward Jenner inoculated a boy with material taken from a lesion on the hand of a milkmaid, who had acquired the infection from cattle. This infection was assumed to be cowpox virus (CPXV), although proof of this is lacking. Jenner subsequently challenged the boy by variolation, but he did not develop smallpox. After additional studies, Jenner published his *Inquiry* that marked the beginning of the vaccination era [6]. In the early 19th century, this method of vaccination (*vacca*, Latin for cow) became very popular because it achieved the same level of protection as variolation coupled with a considerably lower risk of adverse effects. However, limited supplies of the vaccine did not allow widespread vaccinations. To overcome these shortages, vaccine virus was passed by arm-to-arm transfer in humans during the 19th century. A further step towards vaccination success was the production of CPXV and later of the related vaccinia virus (VACV) in the skin of animals. Vaccine stocks were also mass produced on the chorioallantoic membranes of embryonated eggs. No particular VACV strain was officially recommended, but the WHO advised that either the Lister or the New York City Board of Health strain should be used. A further advance was the development of lyophilisation techniques for vaccines in 1950. The availability of such lyophilised virus abolished the need for refrigeration and massively simplified the transportation possibilities to remote areas. Finally, the bifurcated needle allowed even untrained personnel to conduct vaccinations successfully. Recently, it has been stated that there is a potential role of horsepox virus in the origin of the smallpox vaccine [7]: the core genome of a vaccine virus, which had been produced in 1902, has the highest degree of similarity (99.7%) to horsepox virus [8]. This analysis provides evidence of the suspected role of horsepox in the origin of the smallpox vaccine and strengthens the hypothesis that the horsepox virus may be the ancestor of the vaccinia lineage.

## 4. The WHO Smallpox Global Eradication Programme

In 1959, the twelfth World Health Assembly adopted a resolution to achieve the eradication of smallpox [9], however, progress was slow. In 1967, the Intensified Smallpox Eradication Programme started with a more stringent line of action and ring vaccinations were conducted: the identification of new cases was enhanced by the training of health care workers, smallpox patients were isolated, and close contacts were identified and subsequently vaccinated and quarantined. Within a decade, smallpox was eradicated, with the last natural case in Somalia in 1977. On 8 May 1980, smallpox eradication was declared officially by the WHO. Success relied on the following key determinants: (i) smallpox is exclusively restricted to humans (there is no animal reservoir), (ii) VARV cannot establish either latent or persistent infections, (iii) smallpox was easily recognized and contacts could then be vaccinated, (iv) the vaccine induced long-lasting protective immunity as a single dose, the vaccine was easy to apply, and a rather low vaccination dose was required, (v) vaccine stocks could be easily produced within few days, and (vi) the major reaction (“take”) allowed for a fast, easy, and reliable evaluation of vaccination efficiency even by untrained personnel. In 1996, the World Health Assembly passed a resolution that the remaining stocks of VARV should be destroyed and a date for destruction was set for 1999. However, this date passed without destruction taking place and 20 years later the virus stocks endure. During this period, the WHO has authorised research that is essential for public health benefit to take place. This work aims to develop diagnostic tests, antiviral drugs, and safer vaccines.

## 5. Methods for the Diagnosis of Smallpox

Only a combination of rapid diagnosis, followed by the subsequent isolation of patients, vaccination, and quarantine of contacts will be successful in case of a smallpox outbreak [9]. Most critical is the early diagnosis of a patient since any delay would lead to an increase in the number of infected patients. In this regard, it is important to bear in mind that patients can already spread the virus before the rash becomes apparent. The main requirements for laboratory testing are speed combined with high sensitivity and specificity. Various real-time (RT)-PCR combinations had been published for the detection and species-specific identification of OPVs [10,11,12]. Within the United States, one RT-PCR assay that detects VARV specifically has been approved by the Food and Drug Administration, and a method allowing for the simultaneous differentiation of VARV, monkeypox virus (MPXV), CPXV, and VACV in the same RT-PCR mixture [13] was approved in Russia in 2016.

The recent development of portable RT-PCR machines and lyophilized reagents [14] raise the exciting prospect of these techniques being used for rapid diagnosis in the field. The GeneXpert platform (Cepheid Sunnyvale, CA, USA) has been used in Africa to identify cases of human monkeypox. Point-of-care assays are very useful to confirm human infections rapidly, thereby allowing to us initiate medical countermeasures in a timely manner. However, new OPVs identified recently underline the importance that current assays are routinely re-validated. A “state of the art” RT-PCR strategy is a combination of (i) a generic OPV-assay and (ii) a VARV-specific assay. The parallel application of different detection formats, like 5’ nuclease and hybridization probes, can be beneficial. In addition, an internal control to monitor inhibition has to be included. A modified VARV-DNA as a positive control to rule out cross-contamination is of advantage. By following this strategy, VARV can be detected reliably, even in mixtures of VARV and non-VARV OPVs. Extensive genomic sequence analyses of 45 different VARVs revealed a very high degree of nucleotide sequence conservation among these strains; this suggests that there is probably very little difference in the isolates’ functional gene content, increasing the likelihood that sequence-based detection methods will efficiently identify re-emerging VARVs as well. In addition, the low sequence diversity is reassuring and important from a biodefence perspective because it suggests a high probability of identifying VARV infections if tracking single or multisource outbreaks.

## 6. Vaccines against Smallpox

A variety of VACV strains have been used in different parts of the world [9]. Most of the vaccines were propagated on the skin of animals—mainly calves, but also buffaloes, sheep, and rabbits [15]. The safety record was not ideal since a number of adverse effects occurred after vaccination [16,17]. Accidental infections and severe disease progression in individuals with eczema or immunological deficiency were major complications. In a small percentage of vaccinees, severe neurological disorders, such as encephalitis were reported [18]. Changing the process of vaccine production (1st to 2nd generation) enabled a reduction in possible risks related to the production, however, adverse events associated with the vaccine strain were not expected to change.

In response to the risk of an intentional release of VARV, some countries replenished their vaccine stocks [19]. These stocks had been produced in cell cultures or in calves. It is likely that these vaccines will have the same efficacy. Adverse effects are rare but significant. Since genetic heterogeneity has been determined for the Lister and Dryvax vaccines [20,21], second generation vaccines are plaque-purified clones. Their properties are comparable to their parental strains. In 2007, after plaque-purification and extensive testing, ACAM2000 was licensed for use in the United States [22].

Due to significant adverse events following vaccination with replicating VACVs, research resulted in smallpox vaccines based on highly attenuated VACV strains that (largely) do not replicate in humans after vaccination. Modified vaccinia Ankara (MVA) is a VACV strain derived from chorioallantois vaccinia Ankara with a history of 570 passages in chick embryo fibroblasts [23]. As a result, MVA has a restricted host range and is unable to replicate efficiently in human cells. However, most of the viral proteins are expressed [24,25]. MVA had been licensed as a vaccine in Germany and is considered an effective and safe vaccine based on its robust immunogenicity in the absence of virus replication in vivo. Experimental evidence for this was provided by studies with immunodeficient monkeys [26] and mice [27]. Two doses of MVA are needed to induce a robust immune response comparable to a single dose of replicating VACV. However, a single dose of MVA is already protective in animal models and an earlier onset of protection is seen in non-human primates [28]. Even post-exposure protection was demonstrated in mice [27,29]. The approval of MVA was also supported by safety studies in subjects with low CD4 counts. Mild to intermediate atopic dermatitis included a phase 3 study [30]. Based on these investigations, Imvanex^®^ was licensed in all member states of the European Union and in Iceland, Liechtenstein, Canada, and Norway in 2013. In 2019, the vaccine termed Jynneos^®^ was approved by the US FDA for protection against VARV and MPXV. The indication in adults is for active immunization against smallpox. In the USA, a pre-Emergency Use Authorization would allow the use of Imvamune^®^ in immunocompromised persons. MVA is now part of the US Strategic National Stockpile with an initial order of about 30 million doses. This would allow for the pre-event vaccination of first-line responders—a prerequisite in emergency preparation should an unexpected smallpox outbreak scenario occur. 

LC16m8 is a VACV strain that was developed by continuous passage of VACV strain Lister in primary kidney epithelial cells at 30 °C. In 1975, it was licensed in Japan [31,32]. Inoculation is followed by the formation of a so-called vaccine “take” similar to the Lister strain; however, the vaccine strain is temperature restricted, has a limited host range, and adverse effects are significantly reduced [33]. There are no large deletions in its genome, and the majority of the open reading frames are functional, an exception being the *B5R* gene [34] that encodes a glycoprotein that becomes part of the extracellular virus outer envelope [35] and against which neutralizing antibodies are directed [36]. LC16m8 has been stockpiled in Japan, and 80 million doses could be manufactured per year. LC16m8 is a suitable vaccine to be used singly or in combination with first- and second-generation vaccines. 

## 7. Anti-Smallpox Chemotherapeutics

Cidofovir (Vistide) is an approved drug for the treatment of HCMV retinitis in HIV-patients and is also effective against poxviruses. Cidofovir is a nucleoside analogue that selectively inhibits the viral DNA polymerase and reduces the replication of VARV in vitro [37]. Only when given before the onset of rash can cidofovir prevent mortality. However, cidofovir can cause significant nephrotoxicity. In contrast, brincidofovir, the lipid analogue (CMX001) of cidofovir, is available by the oral route, and no nephrotoxicity has been reported [38,39]. Brincidofovir is active against double-stranded DNA viruses, including OPVs. It was effective in an intradermal rabbitpox virus model and in the intranasal ectromelia virus model in mice. On the basis of these results, it is predicted that concentrations of brincidofovir needed for treatment of smallpox can be achieved with those doses that are being evaluated. Tablet and liquid formulations are under development, however, brincidofovir is not yet approved for the treatment of OPV infections.

SIGA Technologies developed TPOXX (tecovirimat, ST-246) for the treatment of smallpox and has already supplied two million treatment courses to the US Strategic National Stockpile. The drug targets the virus F13 phospholipase and acts as an inhibitor of virus egress and blocks the formation of enveloped forms of OPVs, thereby effectively inhibiting virus dissemination both in vitro and in vivo. The US FDA Animal Rule guided TPOXX drug development, as clinical trials are impossible to conduct due to ethical concerns. TPOXX efficacy, against a wide range of OPVs, was demonstrated in several animal models, including VARV in non-human primates [40,41]. The drug has already been applied for the treatment of adverse effects following smallpox vaccination. In May 2018, the US FDA Antimicrobial Drug Advisory Committee voted in favor of TPOXX benefit versus risk, and in July 2018, the FDA approved TPOXX for the treatment of smallpox. Studies have shown that there is no influence on the efficacy of a Dryvax and ACAM2000 vaccination by TPOXX treatment being given at the time of vaccination [42,43]. The licensure of TPOXX represents a remarkable achievement: it is the first drug licensed against smallpox, the first drug licensed against an extinct disease, and the first drug licensed for human use that relied entirely on efficacy data in animals.

The availability of at least two antiviral drugs that work by different molecular mechanisms has been recommended by the US Institute of Medicine. Continued research on antiviral compounds is highly desirable because the acquisition of drug resistance cannot be excluded and mutations that mediate the resistances of different OPVs were reported both for cidofovir [44] and TPOXX [41,45]. This is especially important in light of potential biothreats by synthetically-produced VARV with the intentional introduction of resistance mutations. It also underlines the dilemma of the scientific community in this regard, as general knowledge about resistance mechanisms is vitally important but their publication poses the risk for malignant misuse. 

## 8. Variola virus Evolution

In 2006, the genomic sequences of 47 geographically distinct VARV isolates were reported [46]. The sequences provide insight into VARV genetics, evolution, relationships with other OPVs, and co-evolutionary interactions with the human host [47]. These sequences, together with the known gene sequences and the drug sensitivities of other OPVs, provide evidence that all VARV strains are sensitive to antiviral drugs such as TPOXX and the cidofovir derivative brincidofovir. The genomes of VARV isolates are closely related to each other, suggesting a slow evolutionary drift in poxviruses based, in part, on the high fidelity of the poxviral DNA-dependent DNA polymerase. The available genomic sequencing data show two major clades, one of which contains two subgroups [46,47,48]. Members of the first group include isolates of variola major from Asia that are associated with high mortality, and isolates from Africa that are associated with varying mortality rates. The second group consists of variola minor (alastrim) from South America, which has lower fatality rates. Closely related isolates from the third group are from Western Africa and are associated with intermediate levels of disease severity.

Recently, VARV-DNA was detected in a mummy in Lithuania dating back to 1643 to 1665. A draft genome could be assembled [49] with almost identical fragmentation of various genes, as seen in VARVs isolated in the 20th century. The authors conclude that the loss of gene function seen in modern VARVs compared to some other OPVs must have occurred before 1650 approximately. By calculating phylogenetic trees, the Lithuanian sequence fell basal to all VARV sequences [46,50]. Further analyses revealed that the time scale of VARV evolution is more recent than often supposed— the two major viral lineages diverged within the 18th and 19th centuries. Such a phylogenetic pattern strongly suggests that both clades experienced a major population bottleneck at this time, which most likely lead to the extinction – possibly due to widespread vaccination - of several unsampled older lineages. An example of an extinct European lineage is represented by a VARV genome obtained from human tissue kept in a museum in Prague dating back 160 years [46]. 

## 9. Genomic Manipulation of Orthopoxviruses and Synthetic Biology

Gene synthesis has been used for the de novo synthesis of infectious poliovirus, and of the 1918 pandemic strain of influenza virus [51,52]. The possibility of synthesizing any DNA sequence can enable live OPVs to be reconstructed as well, although this is more challenging given the larger size of poxvirus genomes. In 2002, infectious VACV was recovered after insertion of a full-length viral genome into a bacterial artificial chromosome [53]. Furthermore, transfecting mixtures of VACV DNA fragments and cells that had previously been infected with a helper leporipoxvirus led to recovery of infectious VACV [54]. In 2018, the recreation of an OPV was demonstrated using only publicly available sequence information: several DNA fragments of approximately 30 kilobase pairs that collectively represented the entire horsepox virus genome were purchased and introduced collectively by transfection into cells infected with a leporipoxvirus, and infectious horsepox virus particles were isolated thereafter. The virus was grown, sequenced, and characterised and was found to have the predicted genome sequence and the growth properties described for the horsepox virus [55]. The effort cost approximately 100,000 USD and took about six months. During this period, the primary limiting factor was the length of time required for DNA fragment synthesis to take place in a commercial company. This demonstration of what was known to be possible, increases the potential re-creation of VARV: even if all existing VARV stocks, including those at the WHO collaborating centres, and the potential clandestine stocks were destroyed, the threat of a re-emergence of infectious VARV cannot be ruled out.

*De novo* synthesis of a viable VARV is only one option for creating VARV or a VARV-like virus. There are other methods available to modify the virulence and host-range pattern of any OPV, including VARV, such as homologous recombination. This method is routinely used in basic and applied research. By this method, drug-resistance markers could be introduced into drug-sensitive viruses. The insertion of genes coding for immunoregulatory active proteins interfering with the host immune system can alter virulence properties. This was demonstrated [56] by inserting the interleukin-4 gene in the genome of ectromelia virus, thereby increasing virus virulence significantly. OPV genes can be replaced by others, genes unique to pathogenic OPVs can be inserted into VACV, the homologous parts of one genome could be replaced with synthetic segments copied from other OPVs, or chimeric viruses (for example, a hybrid of MPXV and CMLV) can be generated with potentially novel virulence patterns comparable to those of VARV.

## 10. Orthopoxviruses as Biothreat agents and Biorisks

According to Ken Alibek, a former deputy director of the former Soviet Union’s bioweapons program, the former Soviet Union expanded its bioweapons research program during the 1980s and was eventually able to weaponise smallpox [57]. However, very little information is available about the extent and outcome of these activities. Today, a concern remains that somewhere, somehow, VARV might be kept illegitimately in clandestine stocks. In a rapidly changing world, the impact of an intentional release of VARV would result in a public health emergency of global concern. This concern stems from the facts that smallpox vaccination programmes were stopped decades ago and so an increasing proportion of the world’s population is immunologically naïve for orthopoxviruses, that the percentage of immunosuppressed individuals has increased, and intercontinental air travel allows rapid viral spread around the world. Shedding the virus from the oropharynx and skin before a smallpox diagnosis is confirmed is a real concern, even in countries with highly development medical healthcare systems.

With regard to medical countermeasures, it is extremely unlikely that a widespread vaccination programme will be re-introduced. Ultimately, success in controlling any possible future outbreak of smallpox will depend on supplies of medical countermeasures as well as on the following classical methods used in the past: (i) early detection, (ii) the isolation of smallpox patients, (iii) the quarantine of contacts, and (iv) ring vaccination [58]. Today—and this is of utmost importance—with the availability of an antiviral compound, cases can even be treated instantly, whereas the induction of a protective immunity is delayed. Therefore, it is of great advantage that both vaccination and antiviral therapy can be given simultaneously without a negative impact. 

In the past, smallpox was relatively easy to recognise. Today, health care professionals have to be educated about the characteristics of smallpox, which can easily be confused clinically with monkeypox, or even chickenpox caused by varicella-zoster virus, a herpes virus. The disease monkeypox is caused by monkeypox virus (MPXV), a zoonotic OPV endemic in the rainforests of central and western Africa [59]. Since the global eradication of smallpox in 1977, monkeypox has been considered to be the most important OPV infection in humans. Based on epidemiological data, it is assumed that MPXV is less transmissible among humans than VARV and is also less deadly (case fatality estimates for monkeypox range between 2% and 10%) and that infection could not sustain itself in the human population. Until recently, infections of monkeypox in humans had mostly been limited to central and western regions of Africa. However, during the ongoing monkeypox outbreak in Nigeria, increased human-to-human transmission has been observed and the wide geographic spread, predominantly in urban areas, has raised concerns about the disease. The recent cases of monkeypox observed in the UK [60], Israel [61], and Singapore all originated from Nigeria, demonstrating that the virus is no longer only a concern in Africa.

Beside these known challenges, novel zoonotic poxviruses are being discovered regularly, many of them with zoonotic characteristics like the Alaskapox virus [62] and the Akhmeta virus [63]. It is fair to expect that more examples may follow and any novel poxvirus should be critically assessed for its zoonotic ability and potential to adapt to humans as a host.

Taken together, the range of risks and biothreats caused by OPVs is particularly broad, and constant vigilance is needed in many different sectors of public life, including the public health service, political decision making, and the surveillance of biomedical research and the biomedical industry, when it comes to synthetic biology.
